# Autonomous Detection and Classification of PI-RADS Lesions in an MRI Screening Population Incorporating Multicenter-Labeled Deep Learning and Biparametric Imaging: Proof of Concept

**DOI:** 10.3390/diagnostics10110951

**Published:** 2020-11-14

**Authors:** David J. Winkel, Christian Wetterauer, Marc Oliver Matthias, Bin Lou, Bibo Shi, Ali Kamen, Dorin Comaniciu, Hans-Helge Seifert, Cyrill A. Rentsch, Daniel T. Boll

**Affiliations:** 1 Department of Radiology, University Hospital of Basel, 4051 Basel, Basel-Stadt, Switzerland; daniel.boll@usb.ch; 2 Siemens Healthineers, Medical Imaging Technologies Princeton, Princeton, NJ 08540, USA; bin.lou@siemens-healthineers.com (B.L.); darrylbobo@gmail.com (B.S.); ali.kamen@siemens-healthineers.com (A.K.); dorin.comaniciu@siemens-healthineers.com (D.C.); 3 Department of Urology, University Hospital of Basel, 4051 Basel, Basel-Stadt, Switzerland; christian.wetterauer@usb.ch (C.W.); MarcOlivier.Matthias@usb.ch (M.O.M.); Helge.Seifert@usb.ch (H.-H.S.); Cyrill.Rentsch@usb.ch (C.A.R.)

**Keywords:** prostatic neoplasms, early detection of cancer, magnetic resonance imaging, deep learning

## Abstract

Background: Opportunistic prostate cancer (PCa) screening is a controversial topic. Magnetic resonance imaging (MRI) has proven to detect prostate cancer with a high sensitivity and specificity, leading to the idea to perform an image-guided prostate cancer (PCa) screening; Methods: We evaluated a prospectively enrolled cohort of 49 healthy men participating in a dedicated image-guided PCa screening trial employing a biparametric MRI (bpMRI) protocol consisting of T2-weighted (T2w) and diffusion weighted imaging (DWI) sequences. Datasets were analyzed both by human readers and by a fully automated artificial intelligence (AI) software using deep learning (DL). Agreement between the algorithm and the reports—serving as the ground truth—was compared on a per-case and per-lesion level using metrics of diagnostic accuracy and k statistics; Results: The DL method yielded an 87% sensitivity (33/38) and 50% specificity (5/10) with a k of 0.42. 12/28 (43%) Prostate Imaging Reporting and Data System (PI-RADS) 3, 16/22 (73%) PI-RADS 4, and 5/5 (100%) PI-RADS 5 lesions were detected compared to the ground truth. Targeted biopsy revealed PCa in six participants, all correctly diagnosed by both the human readers and AI. Conclusions: The results of our study show that in our AI-assisted, image-guided prostate cancer screening the software solution was able to identify highly suspicious lesions and has the potential to effectively guide the targeted-biopsy workflow.

## 1. Introduction

Opportunistic prostate cancer (PCa) screening is a controversial topic in the urological literature. Large prostate-specific antigen (PSA)-based screening programs in Europe (European Randomised Study of Screening for Prostate Cancer (ERSPC)) and the U.S. (Prostate Cancer Screening in the Randomized Prostate, Lung, Colorectal, and Ovarian Cancer Screening Trial (PLCO)) were able demonstrate that early diagnosis and early treatment can help to reduce prostate-cancer-specific mortality [1,2]. However, screening programs are associated with relevant rates of overdiagnosis of 27–56% [3] and overtreatment of clinically insignificant cancers [4]. Furthermore, between 15% and 44% of biopsy-proven cancer occurs in patients with PSA levels below 4 ng/mL, which represents the accepted cutoff value to perform prostate biopsy [5]. Omitting biopsy due to low PSA level can ultimately lead to missing clinically relevant cancer. 

For more than a decade, magnetic resonance imaging (MRI) has been established as a powerful tool for prostate cancer diagnosis. The PROMIS study has demonstrated that prostate MRI is a suitable triage tool for biopsy-naïve men, reducing the number of unnecessary biopsies by a quarter while improving the detection of clinically significant cancer [6]. The PRECISION study randomized patients to either systematic biopsies or MRI with no biopsy if MRI was negative, and targeted biopsy if MRI was positive. Targeted biopsies guided by MRI detected significantly more clinically significant cancers while reducing the number of clinically insignificant cancers [7]. Because of these findings, MRI for prostate cancer diagnosis has been integrated into established guidelines [8].

In order to overcome the outlined weaknesses of PSA-based prostate cancer screening programs, the incorporation of the diagnostic strengths of imaging techniques into an image-guided prostate cancer screening, analogous to breast cancer screening, has already been discussed in the literature [9,10]. 

The dilemma of an increased workload due to such an image-guided, opportunistic prostate cancer screening may be addressed with technological advances: (i) shortening scan protocols and (ii) automatization of the image acquisition and reporting processes. There is growing evidence [11,12,13] that biparametric MRI (bpMRI) protocols consisting of T2w and diffusion weighted imaging yield a similar diagnostic performance compared to the conventional multiparametric MRI (mpMRI) approach while reducing scan times in selected patients to as low as 5 min is theoretically possible [14]. Embedding these standardized imaging sequences in automated acquisition and processing environments represents an essential step to make prostate MR imaging a best clinical practice tool for either screening or diagnostic procedures. Concerning the automatization of reporting, artificial intelligence (AI)-supported workflows have been shown to achieve similar performances in detecting suspicious lesions in prostate MRI examinations compared to human readers [15] and to provide valuable assistance if used as a concurrent reader [16]. 

This work focuses on specific cohort: a true MRI screening population consisting of healthy, biopsy-naïve men enrolled in a prospective trial. The purpose of this trial was to evaluate the efforts and resources required to implement a solely bpMRI-based prostate cancer screening program and to employ a state-of-the-art deep learning for detection and classification purposes in order to test the capabilities of this technology for automatization.

Therefore, we investigate whether a deep-learning-based algorithm in combination with biparametric imaging can be used for detection and classification of Prostate Imaging Reporting and Data System (PI-RADS) lesions in asymptomatic men enrolled in this prospective, MRI-based prostate cancer screening trial. We hypothesize that a deep-learning-based algorithm would provide a high-accuracy solution, potentially allowing to integrate this technology in an image-guided screening workflow.

## 2. Materials and Methods

### 2.1. Screening Program and Prospective Trial Information

The study was approved by the local ethics committees (ethics committee Northwest and Central Switzerland; EKNZ 2018-01965, approved: 26 November 2018) and all patients gave informed consent. Participants were prospectively enrolled in a national registered trial (NCT03749993). The primary purpose of this trial is to evaluate the efforts and resources required to implement a solely bpMRI-based prostate cancer screening program. The presented results are part of a post hoc analysis. Participants were included when the following inclusion criteria were met: biopsy-naïve men >45 years with no history of or suspicion for prostate cancer and a life expectancy >10 years. Exclusion criteria comprised the following: acute urinary tract infection, clinical suspicion of severe voiding disorders and/or chronic inflammation of the prostate, and contraindications for MRI examinations. For the present proof-of-concept investigation, participants were eligible for inclusion during a 6-month observation period ranging from December 2018 to June 2019. Within this time period, 49 participants were recruited and enrolled into the study population.

### 2.2. Artificial Intelligence Software Solution

**Figure 1:**
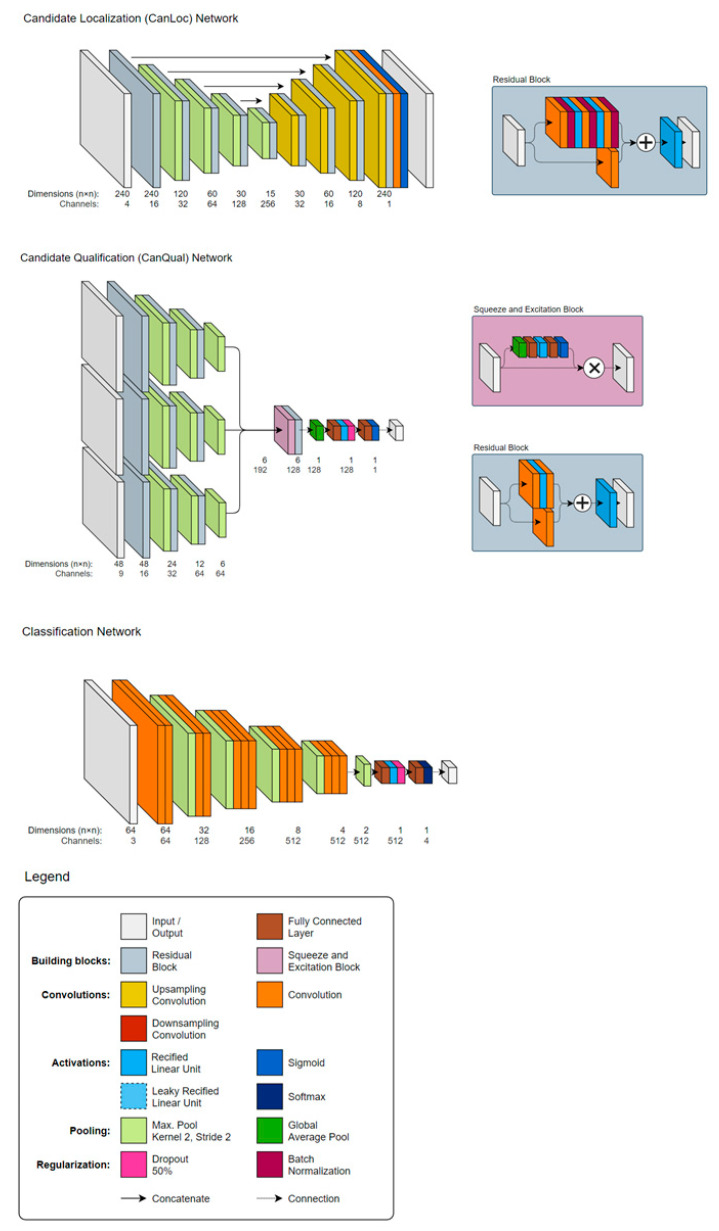
Network architecture of the autonomous Prostate Imaging Reporting and Data System (PI-RADS) lesion detection and classification software solution consisting of a sequence of coupled deep neural networks: a localization network (CanLoc), a candidate qualification network (CanQual)—named false-positive-reduction (FPR) network in Figure 2—and the classification network.

**Figure 2:**
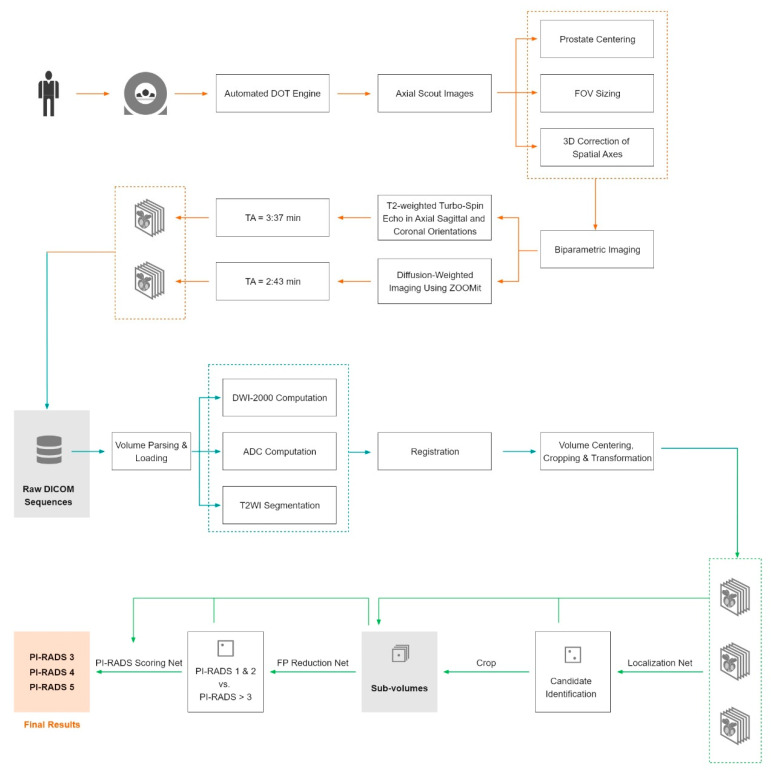
Image acquisition workflow using the automated day optimizing throughput (DOT) engine and biparametric imaging (in orange) as well as the deep learning architecture with the preprocessing pipeline (in blue) and the deep-learning-based lesion detection and classification component (in green). DOT = day optimizing throughput, FOV = field of view, 3D = three-dimensional, TA = time of acquisition, DICOM = Digital Imaging and Communications in Medicine, ADC = apparent diffusion coefficient, FP = false positive, PI-RADS = Prostate Imaging Reporting and Data System.

A deep-learning-based, not commercially available, prototype AI solution (ProstateAI, Siemens Healthineers, Erlangen, Germany, termed in the following text: algorithm) was used for fully automatic prostate lesion detection and classification. A detailed visualization of the network architecture can be found in Figure 1. As illustrated in Figure 2, ProstateAI contains two parts: a preprocessing pipeline and a deep-learning-based lesion detection and classification component. The preprocessing pipeline directly takes the acquired bpMRI sequences and generates the required well-formatted and transformed data volumes. In particular, the preprocessing pipeline first parses and filters the acquired Digital Imaging and Communications in Medicine (DICOM) files loading only the T2w and diffusion weighted imaging (DWI) series. From DWI series, a logarithmic extrapolation method [17] is adopted to compute a new DWI volume with b-value of 2000 s/mm^2^. This step can simultaneously eliminate the b-value variances among the datasets and also improve lesion detection performance [18]. Moreover, apparent diffusion coefficient (ADC) maps are computed. Next, whole-organ gland segmentation is performed on T2w volumes using a learning-based method as presented in [19]. After segmentation, a rigid registration [20] is conducted to align all other sequences (DWI-2000 and ADC) to T2WI.

**Figure 3:**
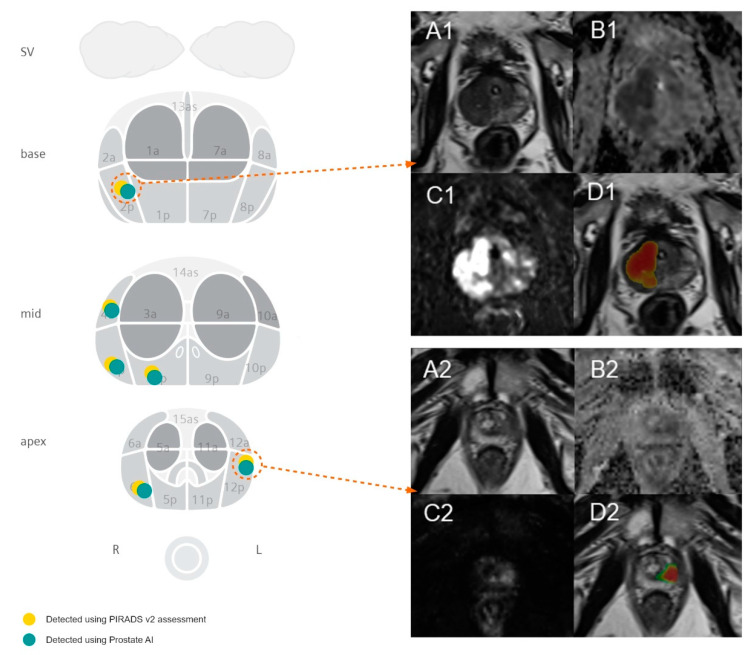
Figure showing the visualization of the automated detection of PI-RADS 5 lesions with positive biopsy results in the peripheral zone in two exemplary cases. The left-hand-side image visualizes the spatial distribution of lesions in our study cohort with a positive biopsy result. Case 1 (**A1**–**D1**) demonstrates a PI-RADS 5 lesion in the left mid-gland PZpl/PZa with a maximum diameter of 35.0 mm and a mean ADC value of 758 s/mm^2^ for 62-year-old men, using the deep learning algorithm. Biopsy results revealed a Gleason 4 + 3 = 7 pattern. Case 2 (**A2**–**D2**) demonstrates a PI-RADS 5 lesion in the right apical PZpl with a maximum diameter of 15.5 mm and a mean ADC value of 961 s/mm^2^ for 51-year-old men, using the deep learning algorithm. Biopsy results revealed a Gleason 3 + 3 = 6 pattern. (**A**) are showing the T2-weighted images, (**B**) the ADC maps, (**C**) the high-b value images (b value of 800), and (**D**) the T2-weighted sequences with overlaying heatmaps displaying the probability of a lesion with red being highly probable and green being less probable as predicted from the algorithm. Both lesions were scored as PI-RADS 5 lesions.

ProstateAI then automatically detects clinically relevant lesions and classifies each detected lesion according to PI-RADS categories. This is achieved by a sequence of coupled deep neural networks that were trained separately. First, a fully convolutional localization net (Candidate Localization Network in Figure 1) is able to generate a semantic lesion candidate heatmap (see examples in D1 and D2 of Figure 3); then, a sub-volume-based, false-positive-reduction net (Candidate Qualification Network in Figure 1) further improves the detection accuracy by removing false positives; last, another sub-volume-based PI-RADS scoring net (Classification Network in Figure 1) stages the level of malignancy for each detection by assigning them to the corresponding PI-RADS categories. The detailed description of the architectures of Candidate Localization and Candidate Qualification Networks can be found in [21].

For this study, the algorithm was trained using 2170 bpMRI prostate examinations consisting of 944 lesion-free cases and 1226 positive cases; all of which had lesion-based PI-RADS information and pixel-based annotations of the lesion boundaries. The anonymized datasets were acquired and labeled at eight different institutions; each institutions’ review board (IRB) provided either exemptions from further review—due to the anonymized nature of the data sets—or full-board IRB approval after review. Using a multicenter approach with standardized reporting, data inhomogeneity for efficient AI training was ensured. Furthermore, we obtained the central processing unit (CPU)-based computational time per case, including the preprocessing and deep-learning component. The CPU-based approach has been chosen in order to most accurately simulate a clinical environment. In this study, we used an Intel^®^ Core™ i7-8850H CPU@2.60 GHz.

### 2.3. MRI Examination

All MRI examinations were performed on a single 3T scanner (MAGNETOM Prisma, Siemens Healthineers, Erlangen, Germany, see Table 1 for a detailed study sequence description). The sequences were embedded into a day optimizing throughput (DOT) workflow, which automatically centers the prostate in the field of view, adapts the size of the field of view, and performs a three-dimensional correction of spatial axes. After coil placement, the DOT workflow does not require further adaptations by technicians while at any time allowing interruptions of the scan process. The total scan time per patient from the start of the first sequence to the end of the last sequence was ~9 min and 30 s, while the scan time for the workflow-relevant series (T2w turbo-spin echo (TSE) tra and DWI) was ~6 min and 20 s. A detailed workflow visualization is outlined in Figure 2. 

All participants underwent a routine clinical reading process in an academic institution with two board-certified radiologists with at least 5 years of experience in prostate imaging reading the cases; at our institution, all prostate imaging studies are read by two independent radiologists as consensus read and the resultant report is highly structured—as suggested by the PI-RADS v2.0 guidelines [22]—including: number of suspicious lesions separated for the peripheral zone (PZ) and transition zone (TZ) with exact locations using series and image number descriptions and reference to the PI-RADS sector map, and PI-RADS score/lesion based on the PI-RADS assessment per zone; especially, the index lesion with the highest PI-RADS score specifically was highlighted per zone.

### 2.4. Histopatholgical Analysis

The biopsies were prepared in the following standardized manner by a uropathologist with more than 3 years subspecialty experience: After fixation in 10% buffered formalin, the biopsy probes were embedded in paraffin wax and sectioned and stained with hematoxylin and eosin according to the pathology committee of the European Randomized Study of Screening for Prostate Cancer. Every biopsy with pathologic prostate parenchyma was attributed a specific Gleason grade on the basis of the underlying glandular pattern. Biopsies with benign prostatic tissue were graded as “normal” if anatomically adjacent cores were tumor free and, additionally, did not show any significant signs of inflammation.

### 2.5. Comparison between Ground Truth and AI

For all lesions with a PI-RADS lesion score of ≥3, targeted transrectal MRI-TRUS Fusion biopsies were performed by a board-certified urologist. Systematic biopsies were not performed. A minimum of 3 cores per lesion were obtained (median number of cores: 3, range: 2–5). 

In order to evaluate the agreement between the ground truth extracted from the written reports with histopathologic correlation and the automatically computed output of the AI software solution, every dataset was manually annotated by a radiology fellow (D.J.W) using a proprietary software (Annotator Tool, V03_B41). The written report contained detailed information about the reported lesions, especially mentioning series numbers on either T2w or ADC with accompanying image numbers and were further visualized as lesions in a PI-RADS sector map. Each lesion was then carefully identified on the DWI series and corresponding ADC maps, using the T2-weighted images as morphological reference. All confirmed lesions were subsequently segmented three-dimensionally on the T2-weighted images in a slice-by-slice fashion and labeled according to the PI-RADS assessment score from 3 to 5 on the ADC map for PZ lesions. In order to evaluate the agreement between the radiologists who identified the lesions in the academic reading process and the annotating fellow, all annotations were reviewed by a senior radiologist (D.T.B.).

The criterion for a true positive labeling of a lesion, defined by the PI-RADS assessment score, was that the detection point of the deep learning software was localized less than 10 mm away from the centroid of the lesion in the annotation in 3D, analogously to (9). False negatives (FN) were defined as lesions annotated by the human reader but not detected by the AI algorithm.

### 2.6. Statistical Testing

Sensitivity, specificity, positive predictive value (PPV), and negative predictive value (NPV) on a case level were calculated based on the algorithm’s results compared to the histologically proven ground truth defined by the radiologists’ reports. A case was classified as positive when a PI-RADS lesions ≥3 was mentioned in the imaging report. Accordingly, a case was evaluated as negative when only PI-RADS 1 and 2 scores were reported. On a per-lesion and index level, sensitivities were calculated. Kappa statistics were applied to compare the agreement concerning the PI-RADS classification. The k coefficients were assessed as follows: 0.01–020, slight agreement; 0.21–0.40, fair agreement; 0.41–0.60, moderate agreement; 0.61–0.80, substantial agreement; and 0.81–0.99, almost perfect agreement. *p* values <0.05 were considered significant. All statistical evaluations were performed using Python (version 3.5, Python Software Foundation; https://www.python.org/).

## 3. Results

**Figure 4:**
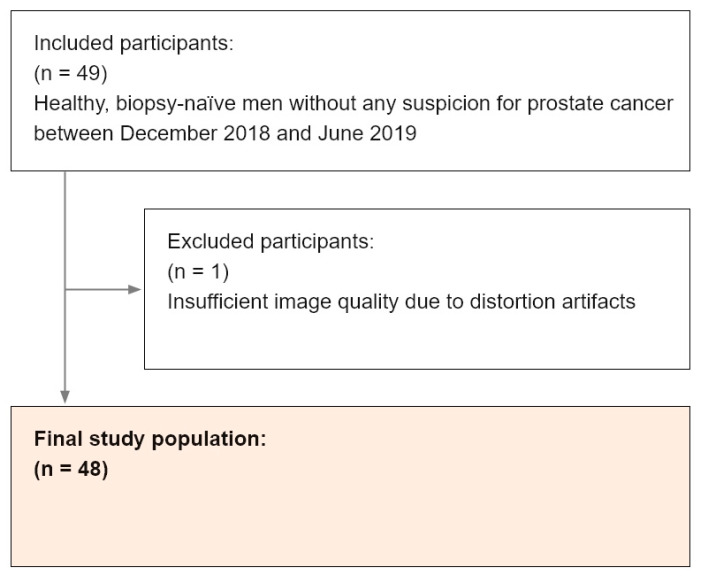
Flowchart outlining the selection of the final study population with utilized inclusion and exclusion criteria within the defined observation window.

From the 49 participants, 1 participant was excluded due to distortion artifacts from the gas-filled rectum and consecutive failure of the image registration between the T2w sequence and ADC map. In total, 48 screening cases were included in the analysis (see Figure 4). The mean age ± standard deviation was 58 ± 8 years (range: 45–75) and the mean PSA value was 2.68 ± 5.48 µg/mL (median: 1.07). The demographic and clinical information is summarized in Table 2. Detailed information concerning the metrics of diagnostic performance can be found in Table 3. The mean CPU-based computational time including image preprocessing, lesion detection, and classification was 14 s per case. All 3D lesion annotations done by the fellow were confirmed by another senior radiologist.

### 3.1. Case-Level Performance

All detected lesions were peripheral zone lesions. With regard to the ground truth, 38/48 (80%) cases had a ≥3 PIRADS score and 10/48 (20%) cases were defined as lesion free based on the radiologists’ reports. The AI solution achieved a case-level sensitivity of 87% (33/38) with a PPV of 87%. The case-level specificity was 50% (5/10) with an NPV of 50%. The case-level k was 0.42.

### 3.2. Lesion-Level Performance

In total, 28 PI-RADS 3, 22 PI-RADS 4, and 5 PI-RADS 5 lesions were assigned in the written reports. Moreover, 22 participants had 1 lesion, 15 participants had 2 lesions, and 1 participant had 3 lesions. The AI solution detected all the PI-RADS 5 lesions (100%), 16/22 PI-RADS 4 lesions (73%), and 12/28 PI-RADS 3 lesions (43%). Overall, 33 of 55 lesions were detected (60%). The mean false-positive rate per patient was 0.875. 

### 3.3. Index-Level Performance

In total, 38 index lesions were assigned by the human readers, according to the total number of positive cases. Of these, 18 index-lesions were classified as PI-RADS 3, 15 as PI-RADS 4, and 5 as PI-RADS 5. The algorithm detected 14/18 PI-RADS 3 (78%), 14/15 PI-RADS 4 (93%), and 5/5 PI-RADS 5 (100%) lesions, resulting in an overall 87% sensitivity on an index-lesion level. 

### 3.4. Biopsy Results

Transrectal biopsies revealed a positive result in histopathology in a total of 6 patients (see Figure 4). Three participants had a Gleason grade group (GGG) of 1 (corresponding PI-RADS scores in these locations were 3, 4, and 5), two had a GGG of 2 (PI-RADS scores: 4 and 5) and one participant had a GGG of 3 (PI-RADS score: 5), see Table 4. All these lesions were detected by the human readers and the AI software solution.

## 4. Discussion

With the ongoing success of deep learning techniques in medical image analysis, those techniques have been used for the detection of prostate cancer. A recent study of Schelb et al. [15], investigating a deep learning system for the detection of suspicious lesions in prostate MRI examinations in men suspected of having clinically significant prostate cancer, showed sensitivity and specificity values of 96% and 31% at a U-Net probability cut-off ≥0.22, and 92% and 47% using a cut-off ≥0.33 in their test set, respectively. The performance metrics in our MRI screening population are very similar, however, our setting is defined by the screening protocol, corresponding to different subject statistics. The total computational time per case in our study was 14 s. It is a known statistical problem that higher sensitivity comes at a price of higher false-positive (FP) rates. A study conducted by Vos et al. [23] showed that at a sensitivity of 74% the FP level was at 5 per patient while the sensitivity dropped to 41% at a FP level of 1. In our study, we opted for a balanced sensitivity and specificity using a FP-reduction (FPR) strategy as the deep learning algorithm tended to overestimate lesions. This approach resulted in a very low false-positive rate per patient of 0.875. Experiments without the FPR strategy yielded sensitivity and specificity values of 97% and 16% on a case level.

The ultimate implementation of such AI-based software solutions in has two prerequisites: (i) agreement between the human reader and the software solution should be non-inferior to human interobserver metrics and (ii) accurate guidance of the biopsy workflow. Concerning the first point, Muller et al. [24] evaluated the interobserver variability of the PI-RADS v2 lexicon in a five-reader study with varying reader experience. The investigators found a k of 0.46 concerning the overall suspicion score. Our results show a similar performance comparing human readers and AI with a k score of 0.42. With regard to the second point, we were able to show that both human readers and the AI solution were able to identify all biopsy-verified prostate cancer lesions. Interestingly, from a screening point of view, only 3/6 participants with a positive histopathology demonstrated PSA values ≥4 µg/mL. In fact, the PSA values demonstrated good capabilities to identify a GGG of ≥2. However, all patients with a GGG = 1 would not have been detected. Therefore, our results may provide new insights in the sense that an MRI-based screening is better suitable as an early warning system. 

We found clinically significant cancers in 3/38 participants (8%). Consequently, in the remaining 35 participants the biopsy did not reveal a clinically significant cancer despite the presence of a PI-RADS lesion ≥3. This may be due to the low tumor yield in PI-RADS 3 lesions in our cohort (3%), potentially warranting a change in the biopsy decision workflow in the future. The PI-RADS score does not equal a cancer identification score but merely is an ordinal, probability score for the presence of cancer. With regard to PI-RADS 3, 4, and 5 lesions, cancer has been detected in the following ranges 12–33%, 22–70%, and 72–91%, respectively [25,26], in studies using extended biopsy sampling schemes. Our values range below these reported values not only because we used targeted biopsies but also due to our distinct study objects, representing healthy individuals with extremely low PSA values and not patients with clinical suspicion for prostate cancer; thus, resulting in a much lower pre-test probability for prostate cancer as compared to a patient cohort with a high level of suspicion for prostate cancer. With regard to the spectrum of pre-test probabilities as published by Lavelle et al. [27], participants in our screening cohort would have been found rather on the “exclusion threshold” for prostate cancer. This fact may explain the slightly lower k values in our screening study compared to data in the literature: as PI-RADS category 3 lesions represented 51% of the reported lesions in our screening cohort (versus a reported incidence of 32% to 22% in patient cohorts [28]) and due to the inherent uncertainty of this PI-RADS category in the guidelines, the detection and classification task presented here is generally more difficult than findings obvious tumor in patients with suspicion for prostate cancer. Larger studies will have to reveal whether the PI-RADS classification scheme can be applied on such a cohort with a certain validity and what management strategies can be developed for those men.

Our study has several limitations. The training data of the algorithm contained more cases with PI-RADS ≥3 lesions than lesion-free cases. Due to this discrepant distribution, the algorithm is somehow prone to a certain over-detection, reducing the overall specificity. However, our specificity ranged between experienced and intermediate/low-experienced readers [29]. Second, all of the lesions under investigation in the current study were peripheral zone lesions, and thus, the results are not valid for transition zone lesions. While reported detection rates of peripheral zone lesions—especially PI-RADS ≥4—are sufficiently high, the detection of transition zone lesions is hampered due to a difficult differentiation of benign and malignant processes [30,31] due to common image features. Here, the capability of artificial-intelligence-based software solutions to detect patterns, which potentially remain invisible to the human eye, hold some promises. Future studies need to investigate the performance of the present or different algorithm(s) in the transition zone. Third, in this cohort we performed targeted biopsies of suspicious lesions only, and no random biopsies were taken. Therefore, it cannot be ruled out completely that lesions not detected in bpMRI and not detected by the algorithm were missed. Fourth, the sample size in our study is rather small. Reasons for this fact are the difficulty to establish a screening population and that the study was designed as a first evaluation of the approach in a proof-of-concept setting.

## 5. Conclusions

In conclusion, this study demonstrates that a deep-learning-based software solution can autonomously detect and classify PI-RADS lesions with a high sensitivity on both a lesion and case level with a moderate classification performance, potentially allowing to use this technology in a screening setting. Furthermore, the AI was able to detect and correctly classify all lesions that contained histopathologically proven cancer, allowing to use that technology in a consecutive targeted biopsy workflow. In an outlook, our approach should be tested in a larger, prospective cohort and the predicted lesions of the AI and the human PI-RADS assessment scores should be compared with the histopathology yield per case and core in a prospective manner.

## Figures and Tables

**Table 1:** Biparametric examination protocol of the prostate.

Parameter	Localizer	T2w tra (TSE)	DWI	T1w tra (TSE)
TA (in min)	0.13	3:37	2:43	2:45
TR (in ms)	3.51	7000	3500	700
TE (in ms)	1.53	104	66	12
ST (in mm)	6	3	3	2
Voxel size (in mm)	1.6 × 1.6 × 6.0	0.3 × 0.3 × 3.0	0.9 × 0.9 × 3.0	0.4 × 0.4 × 3.0
AF	/	3	/	3
b-values (in s/mm^2^)	/	/	0, 800	/

Note that T2-weighted coronal and sagittal orientations were re-sliced from the transversal orientation. TA = acquisition time, TR = repetition time, TE = echo time, ST = slice thickness, AF = acceleration factor, TSE = turbo-spin echo, DWI = diffusion-weighted imaging.

**Table 2:** Demographic and clinical information. PI-RADS = Prostate Imaging Reporting and Data System, PSA = prostate-specific antigen.

Variable	Participants (*n* = 48)
Demographic and clinical information	
Mean age (mean ± SD, in years)	58 ± 8 (range: 45–75)
PSA in µg/mL (median, mean ± SD)	1.07, 2.68 ± 5.48
Algorithm detection rate *	
PI-RADS 1 and 2	5/10 (50)
PI-RADS 3	14/18 (78)
PI-RADS 4	14/15 (93)
PI-RADS 5	5/5 (100)
Positive histopathology results per PI-RADS score	
PI-RADS 3	1/28 (3)
PI-RADS 4	2/22 (9)
PI-RADS 5	3/5 (60)

* Data represent results on a case level with counts for PI-RADS 3, 4, and 5 categories representing the index lesion score per patient and nested PI-RADS 1 and 2 categories if no or clearly benign lesions were reported. Values in parentheses are percentages.

**Table 3:** Algorithm performance on a case and lesion level.

Parameter	Sensitivity (%)	Specificity (%)	PPV (%)	NPV (%)	Kappa k
Case level	87	50	87	50	0.42
Lesion level					
PI-RADS category 3	43	/	/	/	/
PI-RADS category 4	73	/	/	/	/
PI-RADS category 5	100	/	/	/	/
Combined PI-RADS category 4 and 5	78	/	/	/	/

PI-RADS = Prostate Imaging Reporting and Data System, PPV = positive predictive value, NPV = negative predictive value.

**Table 4:** Detailed clinical information for the six participants with histopathologically proven tumor.

Participants	GGG	PI-RADS	PSA (µg/mL)	Maximum Diameter (mm)
1	1	5	3.99	15.7
2	1	3	2.18	6.3
3	2	4	6.47	12.1
4	1	4	0.81	9.05
5	2	5	5.52	21.1
6	3	5	39.70	35.0

The PI-RADS score was derived from the written reports. The PSA value was determined before the biopsy. The maximum diameter of the lesions was derived from the axial slice of the ADC map with the largest tumor diameter. PI-RADS = Prostate Imaging Reporting and Data System, PSA = prostate-specific antigen, GGG = Gleason grade group.
